# Academy of Medicine

**Published:** 1859-11

**Authors:** 


ART. III.—*Academy of Medicine. Contagiousness of Yellow Fever. Fomites.*

An animated discussion took place at the meeting of the Academy of Medicine, on the evening of October 5th, on the contagiousness of yellow fever, and fomites. Most of our readers are probably aware that, at a meeting of the Quarantine and Sanitary Convention in May last—a convention composed of physicians and non-medical delegates—a resolution was adopted, denying the diffusion of yellow fever by contact with the persons of those affected with the disease, but admitting that the disease may be transported by means of fomites. A resolution was submitted to the Academy by the president of the convention, Dr. Griscom, endorsing the action of the latter body. This was opposed by Dr. Reese, who contended that the term fomites was applied only to substances containing a virus generated within the diseased body—in other words, contagious matter; and hence, to say that a disease is communicable from one body to another not directly, but only through the intervention of fomites, is absurd. He denied the communicability of the disease, either directly or indirectly. Dr. Francis expressed his belief in the contagiousness of yellow fever under certain circumstances, but deprecated any decided action of the Academy on the subject, as impolitic, in view of differences of opinion in the profession. Dr. J. M. Smith denied the position assumed by Dr. Reese, with respect to the definition of fomites, regarding the term as denoting the presence, in inanimate substances, of infectious matter of any description, whether generated within or without the diseased body. Dr. Reese rejoined, reaffirming the correctness of his position. Dr. Post, in behalf of the Quarantine

Convention, asserted that, in considering the resolution submitted by Dr. Griscom, the Academy were bound to accept the term fomites in the sense intended by the Convention. The Convention employed the term as applied to substances containing infectious matter not derived from the diseased body. Dr. Mott avowed his belief in the occasional contagiousness of yellow fever.

It will be perceived that the real subject of discussion was the latitude with which the term fomites may be properly used. The discussion was adjourned to the next meeting of the Academy; but from the manifestations of the members present at the meeting, it is not probable that Dr. Griscom's resolution will be adopted.

